# Stiff person syndrome presenting with sudden onset of shortness of breath and difficulty moving the right arm: a case report

**DOI:** 10.1186/1752-1947-4-118

**Published:** 2010-04-27

**Authors:** Bradley Goodson, Kate Martin, Thomas Hunt

**Affiliations:** 1 Department of Psychiatry, University of Nevada School of Medicine, West Charleston Boulevard, Las Vegas, Nevada 89102, USA; 2 Department of Family and Community Medicine, University of Nevada School of Medicine, Fire Mesa Street, Las Vegas, Nevada 89128, USA

## Abstract

**Introduction:**

First described in 1956, stiff person syndrome is characterized by episodes of slowly progressive stiffness and rigidity in both the paraspinal and limb muscles. Although considered a rare disorder, stiff person syndrome is likely to be under-diagnosed due to a general lack of awareness of the disease in the medical community.

**Case presentation:**

A 27-year-old Hispanic woman presented to our emergency department with a sudden onset of shortness of breath and difficulty moving her right arm. Her physical examination was remarkable in that her abdomen was firm to palpation and her right upper extremity was rigid on passive and active ranges of motion. Her right fingers were clenched in a fist. Her electromyography findings were consistent with stiff person syndrome in the right clinical setting. Stiff person syndrome is confirmed by testing for the anti-glutamic acid decarboxylase antibody. Her test for this was positive.

**Conclusion:**

Stiff person syndrome may not be a common condition. However, if disregarded in the differential diagnosis, it can lead to several unnecessary tests being carried out causing a delay in treatment. This case report reveals some of the characteristic features of stiff person syndrome with an atypical presentation.

## Introduction

In 1956, Moersch and Woltman of the Mayo Clinic described an unusual condition of muscle stiffening and difficulty walking. They coined it "stiff man syndrome" [1]. A more appropriate name "stiff person syndrome (SPS)" was later suggested, as the condition affects both sexes, possibly more women than men. Although considered a rare disorder, SPS is under-diagnosed due to a general lack of awareness in the medical community.

Patients with SPS usually experience a prodrome of stiffness and rigidity in the axial muscles of their cervical or lumbar spine. There is a gradual worsening and progression of the condition over time which involves the proximal limb muscles. Pain may be an associated symptom, but significant stiffness and rigidity are the classical features of the disorder. Some symptoms are reported to cause spinal deformities, such as exaggerated lumbar lordosis [2]. Ambulation can be dangerous because the normal postural reflexes of patients become replaced by stiffness, thus placing them at greater risk of fractures. Sometimes, the severity of proximal limb muscle stiffness can overwhelm that of the axial muscles, leading to presenting symptoms of arm or leg rigidity. Such a case is described in this report.

## Case presentation

A 27-year-old Hispanic woman presented to the University Medical Center Emergency Department in Las Vegas, Nevada with a sudden onset of shortness of breath and increased difficulty in moving her right arm. She reported that during the evening prior to her presentation, she was lying down when she began to experience shortness of breath with worsening right-arm weakness. She also reported that for the past two months her arm weakness was characterized as having limited strength and range of motion. She also complained of chest pains that were localized behind her sternum. The pain was characterized as a pressure sensation that was non-radiating. She did not have any aggravating or relieving factors. Pertinent positive findings included nausea, palpitations and lightheadedness. Pertinent negative symptoms included no loss of consciousness, headache, vomiting, diarrhea, or vertigo.

Our patient had been evaluated in the same emergency department two months prior to this presentation for right-arm weakness and dysphasia. During her prior admission to the emergency department, she had received multiple MRI studies of her brain and cervical spine. A previous MRI of her brain had been unremarkable but an MRI of her cervical spine had indicated some mild cervical canal narrowing secondary to end-plate changes and chronic kyphotic changes. At the time of her previous admission, she was diagnosed with hemiplegic migraine headache.

A systemic review of our patient was non-contributory except for an associated dry cough. She denied having a history of headaches or migraine headaches. Her vital signs were stable, and she was afebrile, not tachycardic, not tachypneic, and normotensive. She appeared anxious during her physical examination. She also reported being right-handed. Her cardiac and pulmonary examinations were unremarkable. There were no murmurs on cardiac auscultation noted during her examination, and there were no wheezes, rales or rhonchi while auscultating her lungs.

**Figure 1:**
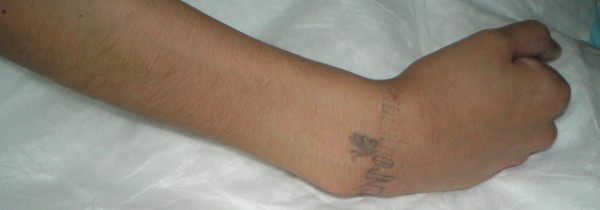
**Image of our patient's right arm demonstrating fingers in a fist-like position**.

**Figure 2:**
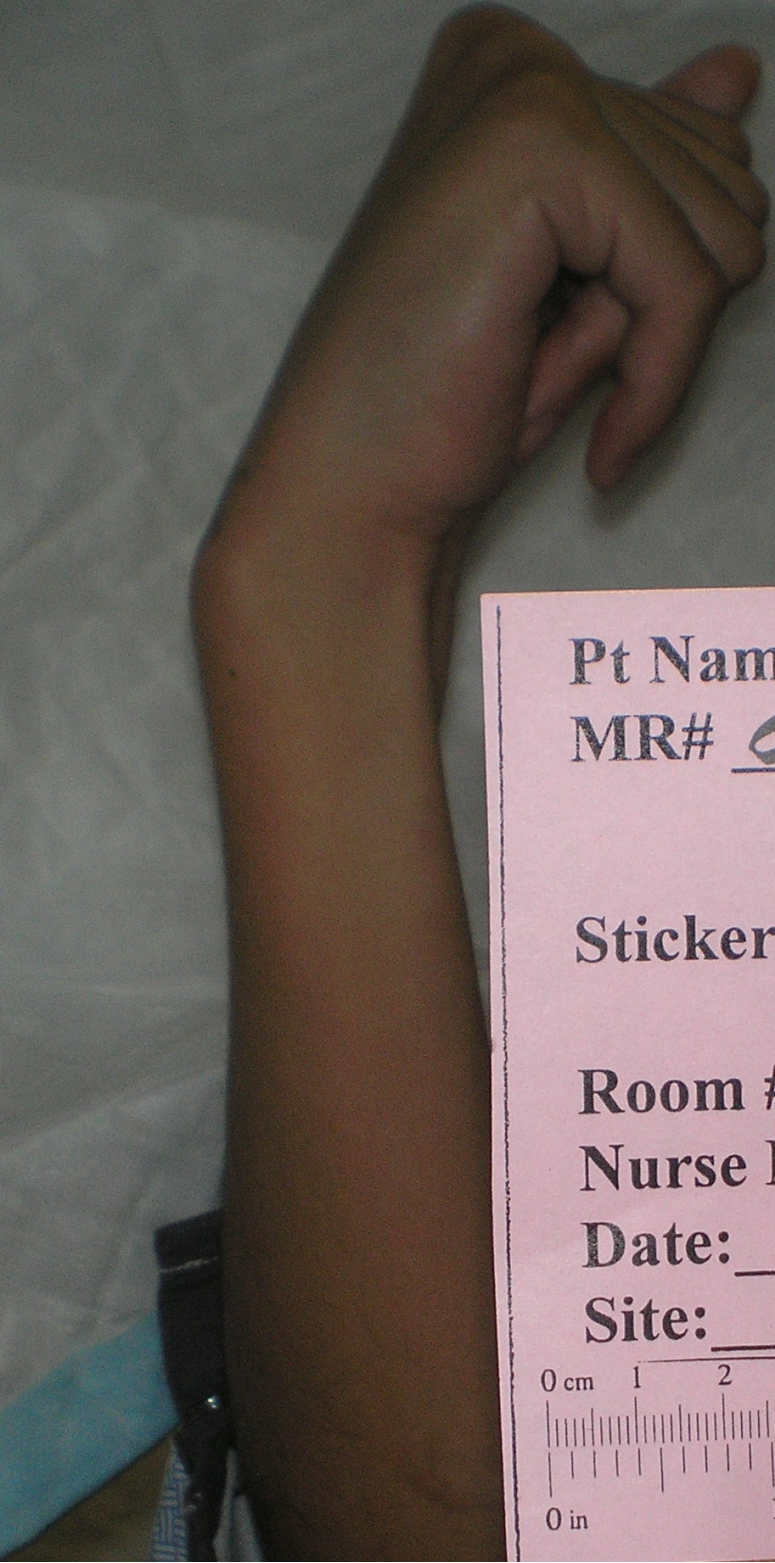
**An image of our patient's right arm revealing spontaneous recoil of fingers to the flexed and fist positions following passive extension**.

Her physical examination was remarkable in that her abdomen was firm to palpation and her right upper extremity was rigid on passive and active ranges of motion. Her right fingers were clenched in a fist (Figure 1). When her fingers were passively extended the digits spontaneously recoiled to the flexed and fist position (Figure 2). Neurologically, she exhibited some dysarthria, but her cranial nerves were intact.

She demonstrated five of the five muscle strength globally, despite exhibiting pain while we were assessing the strength of her right hand. Her deep tendon reflexes were 1+ globally. While testing deep tendon reflexes we noticed that her left leg was unshaven compared to her right leg. She reported that because her hand was constantly in a fist position she had been unable to shave her left leg. The position sensations of both her great toes were intact.

Our patient was placed under observation to rule out acute coronary syndrome. Results of her initial routine laboratory tests and chest X-ray were unremarkable. Because she was complaining of shortness of breath, the emergency room physicians ordered a computed tomography angiogram (CTA) of her chest to rule out pulmonary embolism, and the results came back negative. She then underwent a cardiac stress test and a serial troponin and cardiac enzyme test. Results of her stress test and cardiac enzyme tests were negative. Because of her past diagnosis of hemiplegic migraine headache and her persistent symptoms and dysarthria, a neurology consultation was requested. In addition, because her symptoms did not have an obvious explanation, a psychiatric consult was also ordered to rule out a factitious disorder, a conversion disorder or malingering.

The consulting psychiatrist reported that her symptoms were not due to a factitious or conversion disorder. It was also noted that she was not malingering, and her symptoms were not due to an adjustment disorder. Her level of anxiety was noted as appropriate.

Meanwhile, her consulting neurologist ordered an array of laboratory and imaging studies because her clinical presentation and history did not follow the pattern of hemiplegic migraine. A vascular, rheumatological, coagulopathy, or autoimmune disorder was further investigated, as the etiology for her symptoms for these possibilities could not be ruled out.

To rule out vascular etiology as a cause of her symptoms, she underwent a variety of imaging studies including magnetic resonance angiogram (MRA) of her neck with and without contrast, two separate MRIs of her brain with and without contrast, an MRA of her head without contrast, and a CTA of her head. The results of the imaging studies found no cause for her spastic right upper extremity. However, incidental findings included a germ cell tumor in her pineal region, a narrowing of her left internal carotid artery, and a 9 mm slightly enhancing macroadenoma of the pituitary.

Meanwhile, the following tests were ordered to rule out a coagulopathy: protein C deficiency, protein S deficiency, factor V Leiden, factor II 20210, anti-cardiolipin antibody studies, anti-thrombin III, factor II and fibrinogen levels, and all were all within the normal limits. In addition, results of the following rheumatology laboratory tests were normal: sedimentation rate, C-reactive protein, and rheumatoid factor levels. As for autoimmune laboratory testing, her anti-double-stranded DNA was above the normal limit with a value of 5. Her anti-nuclear antibody (ANA) was positive, and the ANA titer was 1:320 and speckled.

With the above laboratory results and the clinical presentation of our patient, the possibility of an autoimmune disorder was high. Our patient was informed of these findings and their implications. She was started on a medication of one gram Solu-Medrol (methylprednisolone sodium succinate) infused over 24 hours for five consecutive days. On the second day of her five-day treatment, her consulting neurologist also ordered a lumbar puncture and electromyography (EMG). However, the lumbar puncture was unsuccessful because she was unable to keep still during the procedure.

Meanwhile, EMG testing was performed in her right upper extremity muscles, including the dorsal interosseous, pronator teres, pectoral radialis, biceps, triceps, deltoid and opponens pollicis. In all the muscles tested, frequent involuntary runs of motor units (continuous motor unit activity) were identified. Through limb repositioning, her resting activity was studied, revealing absent fibrillations or positive waves. EMG testing of all the muscles involved resulted in normal motor unit morphology and normal recruitment. There was no evidence of myokymic or neuromyotonic discharges. Her EMG findings were consistent with SPS in the right clinical setting. With this information, her Solu-Medrol (methylprednisolone sodium succinate) treatment was discontinued.

The confirmatory test for SPS, anti-glutamic acid decarboxylase (GAD) antibody, was ordered. However, because the test had to be sent to the Mayo Clinic in Rochester, Minnesota, it took two weeks to receive the results. While waiting for the results, our patient was started on the accepted recommended therapy for SPS: baclofen 10 mg orally three times per day for spasticity, as well as intravenous immunoglobulin (IVIG) for five days to increase her immune response. She was also started on Klonopin (clonazepam) for her anxiety and 5/325 oxycodone/acetaminophen for pain.

**Figure 3:**
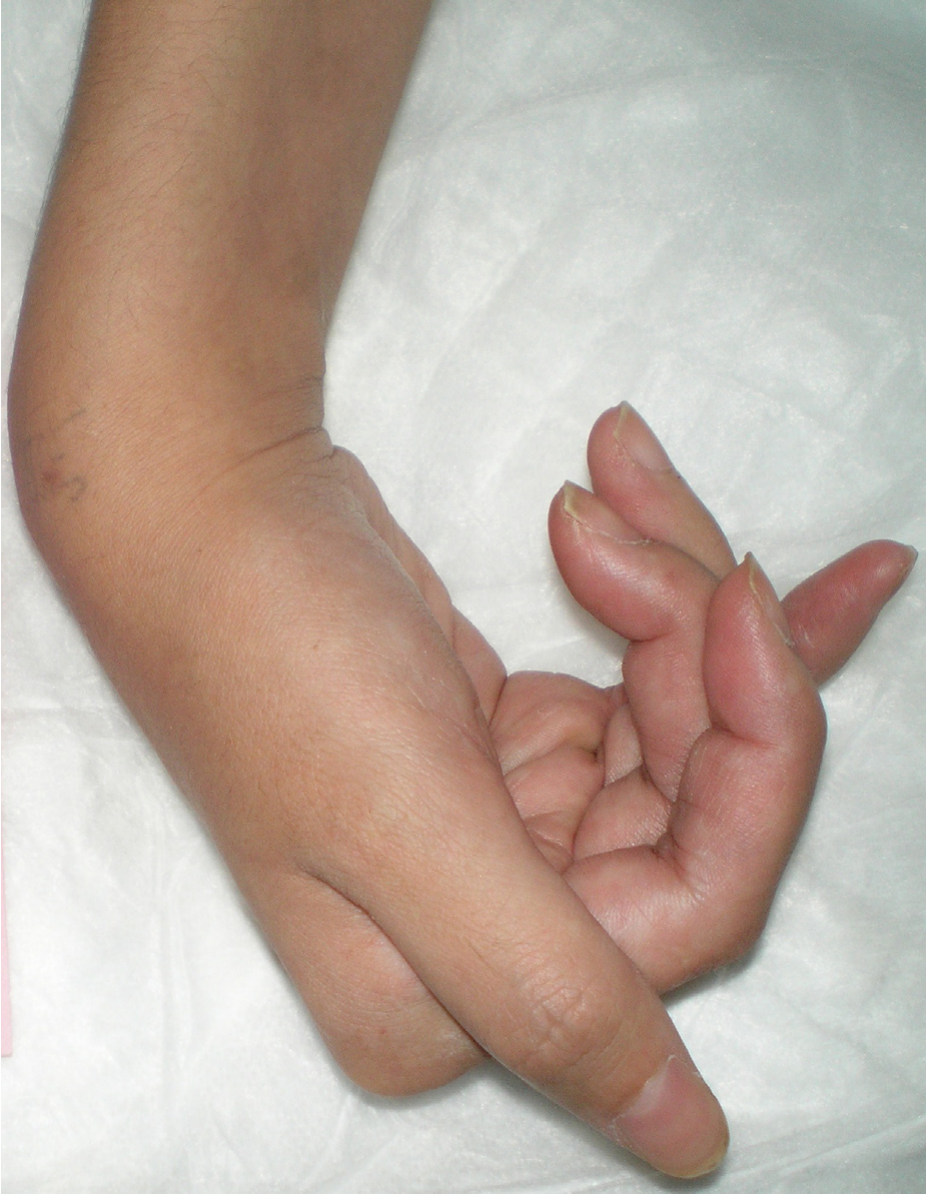
**Example of our patient's ability to actively extend fingers of the right hand five days following the initiation of treatment**.

**Figure 4:**
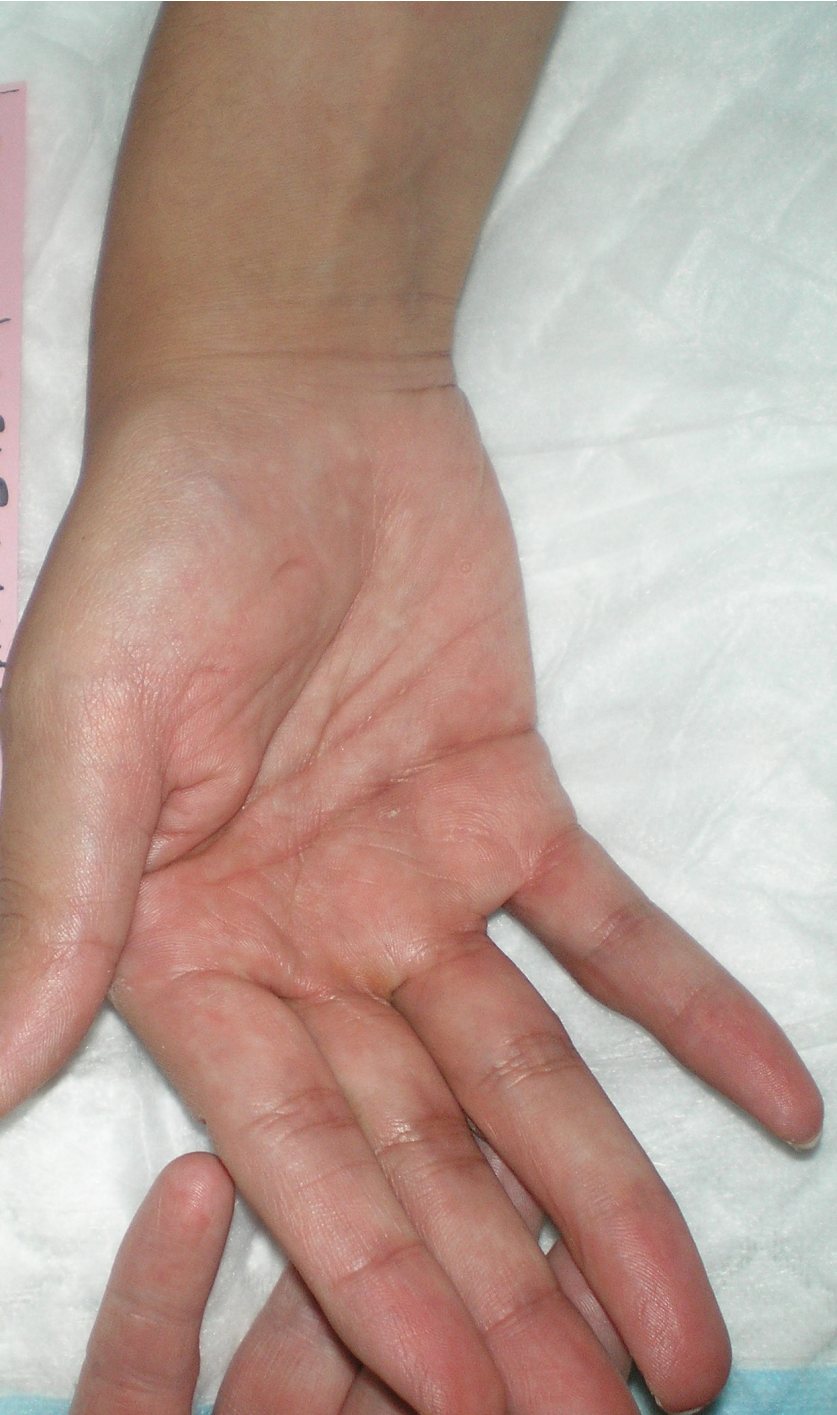
**An image of our patient's right hand following passive extension and without immediate recoil to the flexed position after five days of treatment**.

Over the next five days she began to show clinical improvement. Remarkable changes in her physical examination included a decrease in the spasticity of her right arm, a renewed ability to extend the fingers of her right hand, and an improvement in her dysarthria (Figures 3 and 4). Her abdominal muscles also became less firm. She also received physical rehabilitation while in our hospital. Because of the association between stiff person and paraneoplastic syndromes, the appropriate laboratory investigations for paraneoplastic syndrome were completed, for which our patient's results were all negative.

Upon discharge she was referred to outpatient physical therapy rehabilitation and a neurology follow-up appointment. She also needed to take three medications (baclofen, clonazepam and Percocet). Seven days after her discharge we received the result of her anti-GAD antibody examination, which was positive with a value of 3145 nmol/L (normal range is ≤ 0.02 nmol/L). It is important to point out that the GAD antibody level is not useful as a marker of disease severity or activity, or even as a prognostic indicator. However, it is helpful from a diagnostic standpoint, as in our case. GAD antibody is highly correlated with autoimmune conditions such as diabetes and thyroid conditions. In our case, a thyroid-stimulating hormone was in the normal range, fasting glucose was less than 100 mg/dL, and our patient had no family history of autoimmune disorders. Hemoglobin A1C testing was not performed on our patient, as her random blood sugar levels were less than 200 mg/dL during the time of her hospitalization.

## Discussion

Our case report illustrates an example of SPS with its most prominent manifestation seen in the limb muscles. A key feature of our patient's diagnosis was the occurrence of muscle spasms that were preceded by sudden movement, loud noise or emotional stress, as described in the literature [3]. Specific examples during our patient's hospitalization that precipitated these episodes included being awoken from sleep in the morning and when the medical team entered her room for rounds, as well as an intense fear that made her unable to tolerate a lumbar puncture procedure. Autonomic dysfunction has also been described in the literature. Our patient exhibited some features of this when she had difficulty swallowing, which may have been related to esophageal dysmotility or laryngeal and pharyngeal spasms.

The manifestation of stiffness in an arm, as opposed to the legs or the thoraco-lumbar spine, accompanied by weakness is a peculiar presentation of SPS. However, we feel that the multidisciplinary approach taken to arrive at this diagnosis (neurology and psychiatry) helped us consider many other possibilities.

A recent clinical follow-up on our patient revealed that her symptoms are currently well controlled on a regimen of oral diazepam 7.5 mg twice daily and oral baclofen 20 mg every six hours.

The GAD antibody is found in a number of neurological conditions. One of the main inhibitory neurotransmitters in the central nervous system, gamma-aminobutyric acid (GABA), is regulated by GAD. A decrease in function of the GAD enzyme can lead to less available GABA and, subsequently, heightened stimulation of muscles by motor neurons. The presence of GAD antibodies explains part of this pathophysiological process, because some patients with SPS are GAD antibody-negative. However, GAD is not the only source of GABA. There are other biochemical pathways involved in this disorder that remain to be clarified.

The clinical associations of SPS with other disease processes have been observed, including thyroid disorders, insulin-dependent diabetes mellitus (IDDM) and epilepsy. Much understanding has come from a positive association with GAD antibodies. According to one study [4], IDDM is the most thoroughly documented condition, observed in 25% of patients presenting with SPS. Others cite the figure closer to 60% [5].

Electromyography can be helpful in the diagnosis of SPS, with the detection of continuous motor unit activity, especially in the paraspinal muscles. MRI or CT scanning of the brain is only indicated if there are focal deficits detected on neurological examination, such as abnormal reflexes or frontal lobe signs. However, many patients with SPS will have already undergone extensive imaging as other more common or life-threatening diagnoses were initially being investigated.

First described by Howard in 1963, diazepam is a well-established therapy for SPS [6]. In this case, our patient was initially prescribed the long-acting benzodiazepine relative, clonazepam, and showed an improvement with this medication while she was still in our hospital. Not surprisingly, she also benefited from a muscle relaxant. As in this case, IVIG can be used as an adjunctive therapy in patients with SPS [7]. Although many patients with SPS may not be able to tolerate physical therapy, it was fundamental to our patient's recovery. While weakness is not a typical symptom of SPS, some patients may feel weak and have difficulty with newly regained voluntary movements and fine motor skills.

Rituximab, a monoclonal antibody that binds to the CD20 antigen on B-lymphocytes, has been associated with the long-term remission of SPS, described in a case report from the UK [8]. This report describes a 41-year-old woman with SPS who did not respond to the traditional treatments described above. However, two weeks following the administration of rituximab, her stiffness improved dramatically. Her remaining symptoms were well controlled with low dosages of benzodiazepines, followed by a repeated course of rituximab several weeks later. A phase II clinical trial conducted by the National Institutes of Health investigating the use of rituximab in patients with SPS was recently completed [9]. Although no study results are available at this time, this information may prove helpful to further assess the efficacy of this immune modulator in the treatment of patients with SPS.

## Conclusion

Stiff person syndrome is not a common condition, but it should be considered in the differential diagnosis to avoid unnecessary tests and a delay in treatment. Our case report reveals some of the characteristic features of SPS. However, because of its puzzling presentation, a multi-disciplinary approach helped us reach a correct diagnosis.

## Abbreviations

ANA: anti-nuclear antibody; CT: computed tomography; DNA: deoxyribonucleic acid; EMG: electromyography; GABA: gamma-aminobutyric acid; GAD: glutamic acid decarboxylase; IDDM: insulin-dependent diabetes mellitus; IVIG: intravenous immunoglobulin; MRA: magnetic resonance angiogram; MRI: magnetic resonance imaging; nmol/L: nanomole per liter; SPS: stiff person syndrome.

## Competing interests

The authors declare that they have no competing interests.

## Authors' contributions

TH was the attending physician. KM was a second-year family medicine resident physician and BG was a first-year psychiatry resident physician. BG performed an extensive literature search. Both KM and BG wrote the manuscript. TH reviewed and edited the manuscript. All authors read and approved the final manuscript.

## Consent

Written informed consent was obtained from our patient for publication of this case report and any accompanying images. A copy of the written consent is available for review by the Editor-in-Chief of this journal.
